# Place matters: A longitudinal analysis measuring the association between neighbourhood walkability and walking by age group and population center size in Canada

**DOI:** 10.1371/journal.pone.0189472

**Published:** 2017-12-19

**Authors:** Rania Wasfi, Madeleine Steinmetz-Wood, Yan Kestens

**Affiliations:** 1 Department of Social and Preventive Medicine, École de Santé Publique de l’Université de Montréal (ESPUM), Universite de Montreal, Montreal, Canada; 2 University of Montreal Hospital Research Centre (CRCHUM), Universite de Montreal, Montreal, Canada; West Chester University of Pennsylvania, UNITED STATES

## Abstract

This study examined the influence of walkability on walking behaviour and assessed whether associations varied according to life-stage and population center (PC) size. Walkability scores were obtained for the six-digit postal codes of residential neighbourhoods of 11,200 Canadians, who participated in biennial assessments of the National Population Health Survey from 1994 to 2010. Participants were stratified by age-group. Mixed-effects logistic regression models were used to estimate the influence of cumulative exposure to neighborhood walkability on utilitarian and exercise walking by PC size and life-stage. Associations of neighbourhood walkability with utilitarian and exercise walking varied according to age-group and PC size. Exposure to high walkable neighborhoods was associated with utilitarian walking in younger and older adults in all PC sizes, except for older adults living in a medium PC. Living in a highly walkable neighborhood in a large PC was associated with walking for exercise in younger (OR: 1.42; 95%CI: 1.20–1.67) and older adults (OR: 2.09; 95%CI: 1.51–2.89). Living in highly walkable neighbourhood in a medium PC was associated with walking for exercise in older adults (OR: 1.62; 95%CI: 1.15–2.29). These results emphasize the need to consider the size and nature of every community, and the age-group of a population when implementing strategies to promote walking.

## Introduction

Eighty five percent of Canadian adults do not meet the recommended 150 minutes of moderate to vigorous physical activity per week [1] increasing their risk of contracting chronic diseases such as cardiovascular disease [2], coronary heart disease [3], type 2 diabetes [4, 5], breast cancer [4, 6], and colon cancer [4, 7].

Incorporating walking into the daily routine could be a feasible strategy for adults to meet physical activity recommendations [8] and maintain a healthy body mass index [9]. Walking is a popular form of physical activity that is accessible to individuals of all life-stages and social groups [10]. However, a number of studies suggest that the success of public health strategies that aim to promote walking in the general population will be highly influenced by built environment design [11–13].

Walkability is a central element of built environment design that plays a significant role in influencing the extent to which individuals will engage in walking behavior [14–17]. Studies consistently find walkability to be associated with walking for transportation (also called utilitarian walking) [14, 16, 18–20], while associations between walkability and walking for exercise are mixed [21–24]. Longitudinal studies exploring the relationship in both forms of walking remain scarce [14, 15, 20, 25, 26], limiting causal inference [14, 18].

Findings from longitudinal studies that examine the effect of life-course changes in exposure to the built environment—either through residential relocation or through actual modifications to the built environment itself—on changes in walking can strengthen the evidence base by helping to establish temporal precedence and by controlling for time-invariant confounders such as self-selection variables [27]. Furthermore, recent evidence suggests that associations between walkability and physical activity may vary according to life-stage [28–31] and population center size (PC) (i.e.; towns or cities with a population of ≤1,000 and a density of 400 or more people per square kilometer) [29, 32]. There are a few factors that might explain why associations between walkability and physical activity vary according to population center size. These include variations in how people perceive certain aspects of the built environment, cultural differences between places, or the presence of other physical features that might not be factored into residential neighbourhood walkability scores. A recent study in the United States found that how people perceive certain aspects of the built environment, for example residential density and pedestrian safety may vary depending on whether they live in a small town or in a large metropolitan area, which influenced their walking behaviour [32]. Another study, that examined correlates of leisure time walking for 13,745 participants living in 12 different countries showed curvilinear associations between perceived residential density and land use mix, and adults’ leisure time walking [33]. Evidence from 50 of the largest metropolitan areas in the United-States indicated that road network structure varies across cities. Comparing a set of transportation network structure variables (connectivity, hierarchy, circuity, treeness, entropy and accessibility) suggested that larger cities are physically more inter-connected, which can influence travel behaviour [34]. Longitudinal studies that corroborate these critical findings are paramount.

### Objectives

In this study, our objectives were to (1) examine the association between cumulative exposure to neighbourhood walkability and utilitarian and exercise walking in Canada, and (2) to examine whether these associations vary according to life-stage and population center size.

### Hypothesis

We expect a decline in walking behaviour with age; both for utilitarian purposes and exercise. Moreover, we expect variations in the association between neighbourhood walkability and walking behaviour by life-stage and population center size.

## Methods

Our sample wa**s** obtained from the household component of the National Population Health survey, a nine cycle longitudinal survey of Canadian adults with biennial data collection that occurred between 1994 and 2010. The survey targeted household residents of the Canadian provinces, excluding residents of health institutions, full-time members of the Canadian Forces Bases, those living on Indian Reserves and Crown Lands and those that were living in certain remote areas located in Ontario and Quebec. We restricted our analyses to adults 18 years to 90 years old at baseline, who answered the two following questions on walking behaviour; “*In a typical week in the past 3 months*, *how many hours did you usually spend walking to work or to school or while doing errands*? (*None*, *less than one hour*, 1 *to* 5 h, 6 *to* 10 h, 11 *to* 20 h, *more than* 20 h)” and “have you walked for exercise?”. In order to understand the influence of neighbourhood walkability on walking behaviour across life-stages, we then stratified the sample into two age groups: younger adults aged 18 to 45 at baseline (1994)—i.e., who were 34 to 61 years old at last follow up (2010)-, and older adults aged 65 to 90 years old at baseline—who were 81 or older at last follow-up. During the 16 years of follow-up, some participants never moved while others relocated once or multiple times.

### Measures

#### Outcomes

Self-reported measures of utilitarian walking and walking for exercise were used as outcomes. We reclassified the six categories of walking for transport into two categories, indicating if respondents had engaged in less than an hour (none or low levels of walking) versus more than an hour per week of walking for utilitarian purposes. The threshold of an hour per week of walking was chosen due to the results of an ordered regression model by Wasfi et al. [20] that used the same survey respondents and showed that high walkable neighbourhoods increased the odds of walking for utilitarian purposes for all walking categories that involved walking for at least one hour per week, and decreased the odds of not walking or walking less than an hour a week. For walking for exercise, we created a binary variable that indicated if respondents had reported walking for exercise in the past 3 months.

#### Exposures of interest

Our first exposure of interest was age. To highlight the influence of different life-stages on walking behaviour, age at baseline was modelled as a categorical variable in the model of the entire sample; adults 18 to 45 years old (young adults); 46 to 64 years old (middle age adults); and older adults 65 to 90 years old (65 is the official age of retirement in Canada). Age was used as a continuous variable in the stratified age group models.

Our second exposure of interest was cumulative exposure to neighborhood walkability, i.e., the total number of years spent since 1994 in each walkability category. Walkability was measured using the Walk score^®^, a score varying between 0–100 that is computed based on the distance to different weighted amenities (e.g. shopping centers, schools, parks and restaurants) and has been validated using objective GIS measures of neighbourhood walkability [35, 36]. Walk scores^®^ were obtained for all participants’ residential six-digit postal codes. The five walk score categories identified by walk score.com [37] and used in previous research [38] were re-categorized into 4 categories. The two lowest neighbourhoods walk score categories (0–29) and (30–49) were re-categorized (0–49) into the “low walkability” (LW) neighborhood category, corresponding to all rural areas and the lowest walkable neighbourhoods in population centers (PC). The two highest walk score categories (70–89) and (90–100) were re-categorized (70–100) into the “high walkability” (HW) neighborhood category. The middle walk score category (50–69) was not changed and refers to “medium walkability” (MW) neighborhoods.

For each participant, the cumulative exposure to each neighbourhood type (i.e. walkability category and population center size) was calculated. The measure was adapted from Wasfi and colleagues [20], and modified to take into consideration multiple residential relocations and population center sizes. For each cycle, participants’ residential geographic classification (GC) were defined as rural areas (population<1,000) (RA), small (pop. ≥1,000 and <100,000), medium (pop ≥100,000 and <500,000) or large (pop. ≥500,000) population centers (PC). This resulted in the creation of ten exposure groups; 1) LW in RA; 2) LW in SPC; 3) MW in SPC; 4) HW in SPC; 5) LW in MPC; 6) MW in MPC; 7) HW in MPC; 8) LW in LPC; 9) MW on LPC; and 10) HW in LPC.

Table 1 shows how cumulative exposure time (CET) and the proportion of CET (PCET) were measured, using as an example a person who moved 4 times during the 16 years of follow-up. At baseline, this person resided in a medium walkability neighbourhood (MW) in a medium population center (MPC) and stayed there four years before moving to a rural area (RA-LW) where she stayed another 4 years. She then moved again to a high walkability neighbourhood (HW) in a medium population center (MPC), stayed there another 4 years, and finally moved to a low walkability neighbourhood (LW) in a large population center (LPC).

**Table 1:** Example of cumulative exposure time calculation.

Year	Time (T)	Residential characteristics: walkability category ^1^WC(I) and geographic classification ^2^GC(Z)	^3^CET to RA	CET to MW in MPC	CET to HW in MPC	CET to LW in LPC	^4^PCET to RA	PCET to MW in MPC	PCET to HW in MPC	PCET to LW in LPC
1994	0	MW in MPC	0	0	0	0	0	0	0	0
1996	2	MW in MPC	0	2	0	0	0	2/2	0	0
1998	4	RA**	0	4	0	0	0	4/4	0	0
2000	6	RA**	2	4	0	0	2/6	4/6	0	0
2002	8	HW in MPC	4	4	0	0	4/8	4/8	0	0
2004	10	HW in MPC	4	4	2	0	4/10	4/10	2/10	0
2006	12	LW in LPC	4	4	4	0	4/12	4/12	4/12	0
2008	14	LW in LPC	4	4	4	2	4/14	4/14	4/14	2/14
2010	16	LW in LPC	4	4	4	4	4/16	4/16	4/16	4/16

^1^ WC(I): Walkability category- I ranging from 1 to 3 represented (low (LW), medium (MW) and high walkability (HW))

^2^ GC(Z): Geographic classification—Z ranging from 1 to 4 represented (rural areas (RA), small (SPC), medium (MPC) and large population centers (LPC)).

^3^CET: Cumulative exposure time spent in neighbourhoods with different characteristics.

^4^ PCET: Proportion of cumulative exposure time spent in neighbourhoods with different characteristics.

The proportion of cumulative exposure time (PCET) of respondent X to category I Walkability (WC) and category Z geographic classification (GC) at survey year T = (Total years having resided in WC[I]

* GC[Z])/T, with I ranging from 1 to 3 representing the 3 Walk score^®^ categories (low, medium and high walkability), Z ranging from 1 to 4 representing (rural areas, small, medium, and large population centers) and T ranging from 2 to 16 (in multiples of 2, i.e. 8 follow-ups). For each respondent, the sum of all PCETs values across all walk score categories in different geographic classifications is equal to the number of follow-up years from baseline, and for each follow-up, the sum of PCETs is equal to one.

** Note: rural areas were always in the low walkability category.

Lastly, to understand whether the influence of exposure to neighbourhoods with different walkability scores varied by life-stage, we stratified the sample into two groups younger adults (18 to 45 years old at baseline) and older adults (56 to 90 years old at baseline); to test if they had a statistically significant difference in walking behaviour; both utilitarian and exercise walking.

#### Covariates

All models controlled for time, from 1994 to 2010 (the reference was baseline in 1994). Individual level time-invariant covariates included sex, moving status (i.e., whether they changed their residential location or not during the survey follow up), and immigration status. Time-variant covariates included education, household type and self-perceived health. Retirement status was controlled for in the models to disentangle the effect of age from the effect of being retired on walking behaviour. The language that the survey was administered in and the province of residence was controlled for to account for cultural, lifestyle and weather differences between provinces. Physical activity performed during leisure time was controlled for in models where utilitarian walking was the outcome, to try to isolate the influence of active people selecting to live in walkable neighbourhoods. Moreover, utilitarian walking was controlled for in models where exercise walking was the outcome variable, to isolate any compensatory effect that might have existed because of utilitarian walking. Moving status consisted of the categories non-mover and mover. Education was categorized as follows: did not complete post-secondary education;’ and ‘completed post-secondary education. Self-perceived health was re-categorized into: fair/poor health; good health; and excellent/very good health. Immigration at baseline was re-categorized into: non-immigrant; immigrated less than ten years ago; and immigrated more than ten years ago. Household type was re-categorized into: couple living alone; single person living alone; and couple or single person living with others in the household (e.g. children, family members or friends).

### Statistical analysis

We first modelled our relationships of interest in the entire sample (18 to 90 years old at baseline) and then separately for two age subgroups, i.e., younger (18 to 45 years old) and older adults (65 to 90 years old). We used mixed effects logistic regressions to estimate the effect of cumulative exposure to neighbourhood walkability on both walking outcomes, i.e., utilitarian and exercise walking. Estimating mixed effects regression models allowed us to estimate the effect of age on walking behaviour. All models were conducted using Stata version 14. Transition probabilities from and to each geographic area (LW in RA; LW in SPC; MW in SPC; HW in SPC; LW in MPC; MW in MPC; HW in MPC; LW in LPC; MW on LPC; and HW in LPC) were conducted to ensure adequate sample size in each of the ten categories of residential neighbourhoods.

## Results

### Participants characteristics

Overall we had 11,200 participants 18 to 90 years old at baseline, from which 6,584 were 18 to 45 years old (young adults), 2,699 were 46 to 64 years old, and 1,971 participants were 65 years to 90 years old (older adults). The average attrition rate of respondents (i.e., people who were lost due to death or loss-to –follow up) was 7.8% per cycle.

In the overall sample (adults 18 to 90 years old at baseline), the majority of individuals were women (54.4%), were not immigrants (85.6%), lived with others including children (52.9%), did not complete post-secondary education (69.8%), perceived their health as very good or excellent (60.8%), did not walk for exercise (66.6%), did not walk for more than an hour for utilitarian purposes (58.5%), and were physically inactive during leisure time (61.2%). The average age of the overall sample was (45.5) (SD: 0.15). The majority of respondents responded to the survey in English (81.4%).

The majority of young adults were women (52.7%), not immigrants (88.3%), lived with others including children (71.7%), did not complete post-secondary education (64.8%), perceived their health as very good or excellent (64.8%), did not walk for exercise (67.4%), did not walk for more than an hour per week for utilitarian purposes (55.6%), and were physically inactive during their leisure time (58.0%). The average age of the sample of young adults was 31.9 (SD: 7.7) and 23.8% of young adults moved at least once during the study period.

The majority of older adults were women (61.78%), retired (80.9%), not-immigrants (81.41%), did not walk for exercise (63.11%), did not walk for utilitarian purposes more than an hour a week (63.9%), and did not complete post-secondary education (83.8%). The average age of older adults was 73.6 (SD: 6.1), 46.9% were single and 35.9% of older adults moved at least once during the study period (Table 2).

**Table 2:** Descriptive Statistics at baseline (1994) of the total sample, young (18 to 45 years old) and older adults (65 to 90 years old): National Population Health Survey.

	Total Sample (18 to 90 years old) % or mean (SD)*	Young adults (18 to 45 years old at baseline) % or mean (SD)*	Older adults (65 to 90 years old at baseline) % or mean (SD)
**Sex**			
Men	45.6	47.3	38.2
Women	54.4	52.7	61.8
**Age**	45.5 (0.15)	31.9 (7.7)	73.6 (6.1)
**Immigration status**			
Non-immigrant	85.6	88.3	81.4
Immigrated less than 10 years ago at baseline	3.0	4.4	1.0
Immigrated more than 10 years ago at baseline	11.4	7.2	17.6
**Household type**			
Couple living alone	21.9	14.0	41.7
Single living alone	25.9	14.3	46.9
Living with others including children	52.9	71.7	11.5
**Physically active during leisure time**			
Inactive	61.2	58.0	67.5
Active/ moderately active	38.8	42.0	32.5
**Self-perceived health**			
Poor/fair	12.6	6.1	25.4
Perceived health as good	26.6	23.6	32.7
Perceived health as very good/excellent	60.8	70.3	41.9
**Post-secondary education**			
Did not complete post-secondary education	69.8	64.8	83.8
Completed post-secondary education	30.2	35.2	16.2
Retired	19.24	0	80.9
**Interview administered language**			
English	81.4	80.39	85.17
French	18.6	19.61	14.83
**Utilitarian walking**			
Does not walk or walk <1 hour per week	58.5	55.6	63.9
Walk >= 1 hour per week	41.4	44.4	36.1
**Walking for exercise**			
No	66.6	67.4	63.1
Yes	33.4	32.6	36.9

*SD: Standard deviation.

### Age and retirement status

In the overall sample (18 to 90 years old at baseline), older adults (18 to 64 years old at baseline) were 26% (95% CI: 1.01–1.46) less likely to walk for utilitarian purposes compared to younger adults (18 to 45 years old). No statistically significant difference was found for utilitarian walking when comparing middle aged adults (46 to 64 years old) and younger adults. Every one-year increase in age, was associated with a decrease in the odds of walking for utilitarian purposes by 1% (95%CI: 0.99–1.00) for young adults 18 to 45 years old, and associated with a 4% decrease (95%CI: 0.92–0.95) in the odds of utilitarian walking for older adults (65 to 90 years old) (Table 3). Older adults were also less likely to walk for exercise by 54% (95%CI: 0.39–0.54) compared to young adults (18 to 45 years old). There was no significant difference in the odds of walking for exercise for middle aged participants (46 to 64 years old) compared to young adults (18 to 45 years old). Every decade increase in the age of young adults (18 to 45 years), was associated with a 1% (95% CI: 1.0–1.1) increase in the likelihood of walking for exercise. For older adults (65 to 90 years old) every year increase in age was associated with a 6% decrease in the odds of utilitarian walking (Table 4). In the overall sample, retired adults were 12% (95% CI: 0.8–0.98) less likely to walk for utilitarian purposes compared to non-retired adults (Table 3). On the contrary, retired participants were 20% more likely to walk for exercise compared to non-retired participants (Table 4).

**Table 3:** Mixed effects logistic regression models of utilitarian walking for the total sample, young and older adults: National Population Health Survey (1994–2010).

Utilitarian walking >= 1hr/week (ref. less than 1hr/week)	Total Sample (18 to 90 years at baseline)			Young adults (18 to 45 years at baseline)			Older adults (65 to 90 years at baseline)		
	*Odds Ratio*	*95% C*.*I*.		*Odds Ratio*	*95% C*.*I*.		*Odds Ratio*	*95% C*.*I*.	
**Moved from residence during survey follow-up**	1.05	0.99	1.12	1.08	0.99	1.17	1.10	0.95	1.28
**Women** (ref. men)	1.40**	1.34	1.47	1.50**	1.41	1.60	1.08	0.98	1.27
**Age at baseline** (ref. 18 to 45 years old)									
46 to 64 years old	0.95	0.89	1.02						
65 to 90 years old	0.74**	0.66	0.82						
Age at baseline***				0.99**	0.99	1.00	0.96**	0.95	0.97
**Immigration status at baseline** (ref. not immigrant)									
Immigrated less than 10 years ago	0.80*	0.69	0.91	0.80**	0.68	0.93	0.80	0.40	1.59
Immigrated more than 10 years ago	0.94	0.87	1.01	0.81**	0.72	0.92	0.90	0.77	1.06
**Household type** (ref. couple)									
Living alone	1.05*	1.00	1.12	1.14**	1.04	1.25	1.06	0.94	1.22
Living with others including children	0.98	0.93	1.03	1.02	0.95	1.09	0.88	0.72	1.06
**Leisure time physical activity** (ref. inactive)									
Active/ moderately active	1.35**	1.34	1.4	1.25**	1.19	1.32	1.79**	1.60	2.00
**Perceived health** (ref. poor/fair)									
Good	1.35**	1.26	1.44	1.14**	1.03	1.26	1.69**	1.48	1.94
Very good/excellent	1.46**	1.36	1.55	1.20**	1.09	1.32	2.05**	1.78	2.35
**Education** (ref. did not complete PSE)									
Completed post-secondary education (PSE)	0.99	0.95	1.03	0.96	0.90	1.01	1.12	0.96	1.31
**Retirement status** (ref. not retired) Retired	0.88*	0.80	0.98	-	-	-	0.84*	0.72	0.98
PCET* **to neighbourhoods with different Walk Score**^**^®^**^ **categories in population center (PC) of different sizes** (ref. PCET to rural area)									
PCET to low walkability in small PC	0.93	0.85	1.02	0.94	0.84	1.06	1.09	0.85	1.40
PCET to medium walkability in small PC	1.22*	1.01	1.46	1.23	0.95	1.59	1.14	0.77	1.66
PCET to high walkability in small PC	1.66**	1.31	2.10	1.65**	1.19	2.27	1.67**	1.08	2.57
PCET to low walkability in medium PC	0.78**	0.67	0.89	0.75**	0.63	0.90	0.76	0.53	1.10
PCET to medium walkability in medium PC	0.98	0.83	1.13	1.01	0.82	1.24	0.84	0.60	1.19
PCET to high walkability in medium PC	1.25**	1.06	1.47	1.32**	1.06	1.65	1.32	0.92	1.86
PCET to low walkability in large PC	0.96	0.87	1.05	0.94	0.83	1.06	1.04	0.77	1.39
PCET to medium walkability in large PC	1.23**	1.13	1.34	1.29**	1.15	1.44	1.17	0.94	1.46
PCET to high walkability in large PC	1.61**	1.47	1.76	1.74**	1.56	1.95	1.26*	1.02	1.55
**Language** (ref. English or others)									
French	0.49**	0.43	0.56	0.49**	0.41	0.58	0.54**	0.40	0.74
**Province of residence** (ref. British Columbia)									
Newfoundland & Labrador	0.77**	0.69	0.87	0.89	0.76	1.04	0.44**	0.32	0.60
Prince Edward Islands	0.82**	0.70	0.94	1.02	0.85	1.22	0.50**	0.34	0.72
Nova Scotia	0.95	0.84	1.06	1.11	0.95	1.30	0.64**	0.49	0.85
New Brunswick	0.73**	0.64	0.83	0.82**	0.69	0.97	0.51**	0.37	0.69
Quebec	0.71**	0.60	0.82	0.82*	0.67	1.00	0.51**	0.36	0.72
Ontario	0.99	0.90	1.08	1.09	0.97	1.23	0.82	0.67	1.01
Manitoba	0.84**	0.74	0.95	0.95	0.80	1.11	0.70*	0.52	0.93
Saskatchewan	1.00	0.88	1.13	1.24**	1.05	1.46	0.52**	0.38	0.70
Alberta	0.89	0.79	0.99	1.09	0.94	1.26	0.54**	0.40	0.72
Constant	0.45	0.39	0.52	0.51	0.41	0.63	2.83	1.43	5.62
Random effects variance component:	0.68	0.63	0.73	0.72	0.66	0.78	0.49	0.38	0.63
Number of observations	66,840			40,932			8,980		
Number of participants	11,198			6,584			1,971		
Model fit: AIC	86036.21			52956.77			11147.33		
BIC	86418.83			53301.56			11438.54		

**statistically significant at 99% confidence level.

All models controlled for time, ref. time at baseline

*statistically significant at 95% confidence level.

*** Age was centered around the mean age of the group at baseline.

**Table 4:** Mixed effects logistic regression models of exercise walking for the total sample, young and older adults: National Population Health Survey (1994–2010).

Exercise walking (ref. does not walk for leisure)	Total Sample (18 to 90 years at baseline)			Young adults (18 to 45 years at baseline)			Older adults (65 to 90 years at baseline)		
	*Odds Ratio*	*95% C*.*I*.		*Odds Ratio*	*95% C*.*I*.		*Odds Ratio*	*95% C*.*I*.	
Moved from residence during survey follow-up	1.11*	1.01	1.22	1.16**	1.03	1.31	1.19	0.93	1.51
Women (ref. men)	2.04**	1.89	2.22	2.71**	2.47	2.97	0.99	0.81	1.22
Age at baseline (ref. 18 to 45 years old)									
46 to 64 years old	0.91	0.83	1.00						
65 to 90 years old	0.46**	0.39	0.54						
Age at baseline***				1.01**	1.00	1.01	0.94**	0.92	0.95
Immigration status at baseline (ref. not immigrant)									
Immigrated less than 10 years ago	0.43**	0.35	0.54	0.41**	0.33	0.51	1.33	0.46	3.84
Immigrated more than 10 years ago	0.82**	0.72	0.92	0.66**	0.55	0.79	0.95	0.74	1.23
Household type (ref. couple)									
Living alone	0.79**	0.73	0.86	0.79**	0.70	0.89	1.19	0.98	1.44
Living with others including children	0.80**	0.74	0.86	0.83**	0.75	0.91	0.99	0.77	1.28
Utilitarian walking (ref. None or <1hr/ week)									
Walked for utilitarian > = 1hr per week	1.32**	1.26	1.38	1.22**	1.15	1.30	1.62**	1.43	1.83
Perceived health (ref. poor/fair)									
Good	1.57**	1.45	1.70	1.45**	1.29	1.63	1.79**	1.52	2.09
Very good/excellent	1.98**	1.82	2.15	1.84**	1.63	2.07	2.30**	1.95	2.75
Education (ref. did not complete PSE)									
Completed post-secondary education (PSE)	1.42**	1.32	1.53	1.30**	1.19	1.40	1.48**	1.15	1.90
Retirement status (ref. not retired) Retired	1.19*	1.02	1.39	-	-	-	1.25	0.98	1.59
PCET* to neighbourhoods with different Walk Score^®^ categories in population center (PC) of different sizes (ref. PCET to rural area)									
PCET to low walkability in small PC	1.04	0.92	1.19	1.06	0.90	1.25	0.85	0.58	1.23
PCET to medium walkability in small PC	1.54**	1.17	2.01	1.31	0.90	1.90	1.65	0.92	2.99
PCET to high walkability in small PC	1.39	0.98	1.98	1.20	0.77	1.89	1.73	0.87	3.42
PCET to low walkability in medium PC	1.12	0.94	1.35	1.18	0.94	1.48	0.67	0.42	1.07
PCET to medium walkability in medium PC	0.90	0.75	1.08	0.91	0.71	1.16	0.95	0.64	1.41
PCET to high walkability in medium PC	1.22*	1.00	1.50	1.08	0.83	1.41	1.53*	1.00	2.36
PCET to low walkability in large PC	1.24**	1.07	1.43	1.10	0.93	1.31	0.77	0.49	1.20
PCET to medium walkability in large PC	1.18*	1.03	1.34	1.01	0.86	1.19	1.62**	1.15	2.29
PCET to high walkability in large PC	1.48**	1.30	1.69	1.42**	1.20	1.67	2.09**	1.51	2.89
Language (ref. English or others)									
French	0.83	0.68	1.00	0.70**	0.55	0.90	0.81	0.52	1.28
Province of residence (ref. British Columbia)									
Newfoundland & Labrador	0.70**	0.58	0.86	0.89	0.70	1.13	0.28**	0.17	0.47
Prince Edward Islands	0.61**	0.48	0.76	0.65**	0.50	0.85	0.55*	0.32	0.98
Nova Scotia	0.81**	0.67	0.98	1.07	0.83	1.36	0.54**	0.35	0.85
New Brunswick	0.81**	0.66	0.98	0.93	0.72	1.20	0.62	0.38	1.00
Quebec	0.57**	0.45	0.72	0.64**	0.48	0.86	0.47**	0.27	0.80
Ontario	0.73**	0.63	0.84	0.77**	0.64	0.92	0.65*	0.46	0.92
Manitoba	0.66**	0.54	0.80	0.66**	0.52	0.84	0.65	0.40	1.02
Saskatchewan	0.74**	0.60	0.91	0.79	0.62	1.02	0.72	0.45	1.15
Alberta	0.82**	0.69	0.98	0.90	0.73	1.13	0.79	0.49	1.26
Constant	1.42	1.16	1.74	1.01	0.76	1.34	40.8	14.6	113.9
Random effects variance component:	2.33	2.20	2.46	0.716	0.656	0.780	2.49	2.15	2.89
Number of observations	66,908			40,932			8,980		
Number of participants	11,200			6,584			1,971		
Model fit: AIC	68218.72			52956.77			10236.32		
BIC	68601.39			53301.56			10527.54		

**statistically significant at 99% confidence level

All models controlled for time, ref. time at baseline.

*statistically significant at 95% confidence level.

*** Age was centered around the mean age of the group at baseline.

### Walkability

Adults 18 to 90 years old that lived in residential areas with a high walk score (walk score 70 and above) were more likely to walk for utilitarian purposes, compared to adults living in rural residential areas. This was true for small (OR: 1.66; 95%CI: 1.31–2.10); medium (OR: 1.25; 95%CI: 1.06–1.47) and large population centers (PC) (OR: 1.61; 95%CI: 1.47–1.76). Adults were also more likely to walk for utilitarian purposes if they lived in a large PC with medium neighbourhood walkability (OR: 1.23; 95%CI: 1.13–1.34) or if they lived in a small PC with medium neighbourhood walkability (OR: 1.22; 95%CI: 1.01, 1.46). Adults living in low walkable neighbourhoods in small PC were 22% (95% CI. 0.67–0.89) less likely to walk compared to adults living in rural residential areas (Table 3).

Young adults that lived in residential areas with a high walk score were more likely to walk for utilitarian purposes, compared to younger adults that lived in rural residential areas. This was true for small (OR: 1.65; 95%CI: 1.19–2.27), medium (OR: 1.32; 95%CI: 1.06–1.65), and large population centers (PC) (OR: 1.74; 95%CI: 1.56–1.95). Younger adults were also more likely to walk for utilitarian purposes if they lived in a large PC with medium neighbourhood walkability (OR: 1.29; 95%CI: 1.15–1.44), whereas living in a medium PC with low neighborhood walkability was associated with lower odds of utilitarian walking (OR: 0.75; 95%CI: 0.63–0.90). By contrast, older adults were significantly more likely to walk for utilitarian purposes if they lived in small (OR: 1.67; 95%CI: 1.08–2.57) or large PC (OR: 1.26; 95% CI: 1.02–1.55) in a neighborhood with high walkability, but they were not significantly more likely to walk for utilitarian purposes if they lived in a neighborhood with high walkability located in a medium PC.

In the overall sample, adults 18 to 90 years old that lived in a large population center were more likely to walk for exercise, compared to adults that lived in rural residential areas. This was true for neighbourhoods with high (OR: 1.24; 95%CI 1.30, 1.69), medium (OR: 1.18; 95% CI 1.03, 1.34), and low walkability (OR: 1.24; 95% CI 1.07–1.43) levels. Adults who lived in medium PC and small PC with medium neighbourhood walkability were also more likely to walk for exercise compared to adults residing in rural areas.

Exposure to neighborhoods with high walkability was only significantly associated with walking for exercise in younger adults living in large PCs (OR: 1.42; 95%CI: 1.20–1.67). Older adults were more likely to walk for exercise compared to older adults who lived in rural neighborhoods, if they lived in residential neighborhoods with high walkability located in a large PC (OR: 2.09; 95% CI: 1.51–2.89), if they lived in a neighborhood with medium walkability located in a large PC (OR: 1.62; 95% CI: 1.15–2.29), or if they lived in a neighborhood with high walkability located in a medium PC (OR: 1.53; 95%CI: 1.00–2.36).

### Covariates

In the overall sample, women were more likely to walk an hour or more per week for utilitarian purposes (OR: 1.40; 95% CI: 1.34–1.47) and walk for exercise (OR: 2.04; 95% CI: 1.89–2.22) than men. This was true for both younger and older adults. Younger adult women were more likely to walk an hour or more per week for utilitarian purposes (OR: 1.50; 95% CI: 1.41–1.60) and walking for exercise (OR: 2.71; 95% CI: 2.47–2.97) than men. Individuals who perceived their health as good or very good /excellent were more likely to walk an hour or more per week for utilitarian purposes and walk for exercise in the overall sample and in both age groups separately. Those who were active during their leisure time were more likely to walk an hour or more per week for utilitarian purposes in younger (OR: 1.20; 95%CI: 1.09–1.32) and older adults (OR: 2.06; 95% CI: 1.79–2.36) and those who walked for utilitarian purposes were more likely to walk for exercise in both younger (OR: 1.22; 95% CI: 1.15–1.30) and older adults (OR: 1.62; 95% CI: 1.43–1.84).

Completing post-secondary education was positively associated with exercise walking in both age-groups, but not in the total sample. In most provinces (e.g., Quebec), older adults walked significantly less for exercise and utilitarian purposes than older adults living in British Columbia. After controlling for the language that was used to complete the survey, the odds of walking for utilitarian and exercise purposes for people living in Quebec was still the lowest; but not significantly different from other provinces (i.e., Ontario or Manitoba). People who completed the survey in French reported lower levels of utilitarian walking in the overall sample and in the sample of younger (OR: 0.49; 95% CI: 0.41–0.58) and older (OR: 0.54; 95% CI: 0.40–0.74) adults. Younger adults who completed the survey in French also had lower odds of walking for exercise than younger adults who completed it in another language (OR: 0.70; 95% CI: 0.55–0.90) (Table 2) and (Table 3).

## Discussion

This research makes an important contribution to the literature by showing that living in a high walkable neighborhood is associated with walking for utilitarian purposes in younger adults located in population centers of all sizes, whereas living in a high walkable neighborhood was only associated with utilitarian walking for older adults living in neighborhoods located in small or large population centers. In contrast, results from a cross-sectional study conducted using the Canadian Community Health survey revealed a positive association between the walk score and transport walking for all age groups and for all population center sizes [29]. Sense of pedestrian safety, community attachment and social support could be higher in small population centers compared to large metropolitan areas, which can explain why the odds of walking for utilitarian purposes in older adults is higher in small PCs. Previous longitudinal studies found that walkable environments will both promote [20, 26, 39, 40] and help to maintain levels of utilitarian walking over time [26]. Findings from a residential relocation analysis showed that moving to an area with a 10 point higher walk score was associated with a mean increase of 17.51 minutes of transport walking per week [14]. Similarly, a longitudinal study of Canadians demonstrated that moving to a more walkable neighborhood increased the odds of walking by 59% [20].

Previous research also suggests that walkability may be a determinant of exercise walking [22–24]. However, these studies have used geographically dispersed samples covering a vast array of urban areas without controlling for population center size [22–24]. Our findings indicated that high walkability incited exercise walking but only in large and medium population centers suggesting that there may be other factors associated with small population centers such as social norms that may discourage individuals from walking for exercise in walkable areas, or that promote car use. It could also be a result of differences in how residents perceive neighbourhood walkability in population centers of different sizes.

This study has many strengths that merit consideration. The findings advance emerging literature investigating longitudinal associations between the built environment and walking and is unique in that it permits the generalization of the results to younger and older adults of multiple population centers of various sizes [29]. We also controlled for provinces allowing us to capture some cultural differences and variations in weather that could have otherwise induced bias. Finally, our study is one of few that has examined whether the effect of the built environment on walking varies according to life-stage [28, 30, 31, 41]. Longitudinal evidence examining these potential differences is important, as compared to younger adults, the decisions of older adults will be less dependent on time constraints such as transporting children and strict work schedules [31]. It is also increasingly recognized that the built environment plays a pivotal role in healthy aging [42]. Older adults are a unique sub-population, whose transport behaviour may be especially sensitive to their surroundings, given that overcoming barriers in the physical environment becomes increasingly difficult with age [43, 44].

Our study did not control for self-selection factors such as the participants’ reasons for moving into their neighborhood. This can introduce bias, as those who are physically active and favor walking as a means of transport may be more likely to move into a walkable neighborhood to facilitate their transport behavior. This potential bias was, however, reduced by controlling for leisure time physical activity. Our study used self-reported measures of walking, which can be subject to over or under reporting of walking levels due to social desirability or recall difficulties [1]. However, to an extent self-reporting is less of a problem in longitudinal studies, as under or over reporting is likely consistent over time for a given individual. We were unable to obtain Walk score^®^ data for the all cycles of the study; however, changes to the built environment tend to occur at a slow pace and GIS street network and residential density measures, which are correlated with the walk score, obtained ten years apart (1996 and 2006) for NPHS residential neighborhoods have been found to be highly correlated [20]. Identifying the true location of the residence of those living in rural areas can be imprecise [29]. However, because rural areas were coded with one unique walkability category, equivalent to low walkable neighbourhoods in urban areas (walk score 0–49), it still gave us the opportunity to compare the walking behaviour of individuals living in rural areas to the walking behavior of individuals living in urban neighbourhoods.

The results from our longitudinal analysis indicate that the effect of neighbourhood walkability on walking for transport and walking for exercise varies according to life-stage and population center size. Our findings indicate that utilitarian and exercise walking were significantly correlated with neighbourhood walkability for young and older adults. Increasing the number and type of destinations within walking distance in neighbourhoods could be used as a strategy to promote walking and healthy aging. Public health professionals and city and urban planners should pay attention to the size and nature of every community (places), when they implement strategies to promote walking. Moreover, future longitudinal studies that use GPS trackers, detailed travel dairies and activity space questionnaires could account for daily walking behaviour occurring outside of residential neighbourhoods to further our understanding of how built environments relate to active living.
